# Enhancing the quantification of post-occlusive reactive hyperemia: a multimodal optical approach

**DOI:** 10.1007/s00424-025-03110-7

**Published:** 2025-09-02

**Authors:** Henrique Silva, Carlota Rezendes, Pedro Contreiras Pinto

**Affiliations:** 1 Research Institute for Medicines (iMed.ULisboa), Faculdade de Farmácia, Universidade de Lisboa, Av. Prof. Gama Pinto, 1649-003 Lisbon, Portugal; 2 Department of Pharmacy, Pharmacology and Health Technologies, Faculdade de Farmácia, Universidade de Lisboa, Av. Prof. Gama Pinto, 1649-003 Lisbon, Portugal

**Keywords:** Post-occlusive reactive hyperemia, Videocapillaroscopy, Near-infrared reflectance imaging, Vein finder, Photoplethysmography, Contralateral response

## Abstract

Post-occlusive reactive hyperemia (PORH) is a physiological response marked by a transient increase in microvascular perfusion following ischemia. While cutaneous perfusion during PORH has been extensively characterized using optical approaches such as Doppler-based techniques, low-cost alternatives like photoplethysmography (PPG), videocapillaroscopy (VC) and near-infrared reflectance imaging (NIRI) may provide complementary insights into both microvascular and venous dynamics. However, their role in quantifying PORH remains underexplored. This study aimed to evaluate the potential of low-magnification VC and NIRI-based imaging for quantifying perfusion changes during a standardized PORH protocol in healthy subjects, using PPG as a reference. Fourteen participants (21.5 ± 4.2 years) underwent suprasystolic occlusion of a randomly selected upper limb, with simultaneous recordings using PPG and VC at the finger and NIRI at the dorsal hand veins. The protocol included a 5-min baseline, 3-min occlusion (200 mmHg), and 5-min recovery. Skin blood flow was derived from the PPG signal, a hemoglobin index (C_Hb_) was extracted from VC images, and vein width was measured using NIRI. Nonparametric statistics were used for analysis. Arterial occlusion significantly reduced skin blood flow (–95.3%, *p* < 0.001) and C_Hb_ (–8.3%, *p* = 0.007), with milder contralateral changes. Vein width increased during occlusion (*p* = 0.003) and returned to baseline during recovery. VC was less sensitive than PPG but reproduced the expected hemodynamic profile. A positive correlation was found between venous dilation during recovery and the decrement velocity of microvascular perfusion during occlusion. VC and NIRI represent accessible and complementary tools for assessing vascular responses during PORH. Their combined application may enhance non-invasive vascular evaluation in both clinical and research settings.

## Introduction

Reactive hyperemia is a well-studied physiological response characterized by a transient increase in perfusion following an ischemic event [1]. Experimentally, this response can be easily evoked by the transient occlusion of a limb artery with a pneumatic cuff, thereby termed post-occlusive reactive hyperemia (PORH). Alternatively, a response similar to that of reactive hyperemia can also be evoked by passively raising a limb and rapidly returning it to its original position, with the advantage of being more comfortable for the subject [2]. In PORH, the blood volume accumulated proximal to the occlusion is suddenly released into the previously occluded artery, causing a transient increase in arterial diameter, a phenomenon known as flow-mediated dilation (FMD). This parameter can be assessed with echo-Doppler techniques and has been used in the last decades as a measure of endothelial function in vivo [3]. Conversely, in tissues distal to the occlusion, PORH can be evaluated by measuring perfusion increase using various techniques, such as arterial tonometry, photoplethysmography (PPG), laser Doppler flowmetry (LDF), near-infrared spectroscopy (NIRS), tissue viability imaging (TiVi), or nailfold capillaroscopy (NFC) [4, 5]. While most of these methods offer high sensitivity, their widespread clinical use is limited by cost, complexity, or operator dependency. Among them, PPG and NFC stand out as compact and affordable alternatives.

Nailfold capillaroscopy consists in the direct observation of the cutaneous capillaries through a microscope equipped with an epi-illumination system [6]. When performed at high magnifications (250 × and higher) and used to assess red blood cell velocity in real time, this technique is referred to as videocapillaroscopy (VC). Recently, digital microscopes have significantly reduced costs and improved usability, facilitating broader clinical applications [7]. However, due to their lower magnification capacity, these devices are not well suited for direct flow assessments. An alternative strategy is to analyze image features associated with perfusion, such as changes in color, particularly those related to oxyhemoglobin content (OxyHb). To our knowledge, no previous study has attempted to quantify perfusion during PORH through OxyHb-related color changes in low-magnification VC images.

Although PORH has been extensively characterized in terms of arterial and microvascular responses, the venous compartment also plays a fundamental role in local hemodynamic regulation [8]. Venous adaptations during PORH, however, remain largely unexplored, likely because most studies rely on Doppler ultrasound, which is costly and operator-dependent. Near-infrared reflectance imaging (NIRI) devices, commonly referred to as “vein finders” represent a low-cost alternative capable of real-time visualization of superficial veins [9–11]. Investigating venous adaptations during PORH may shed light on blood volume redistribution, venous compliance, and their interaction with capillary drainage.

From a clinical perspective, accessible and non-invasive methods to assess both microvascular function and venous dynamics are highly desirable, particularly in conditions where early vascular dysfunction plays a central role, such as hypertension, diabetes, systemic sclerosis, and preeclampsia. The present study investigates whether low-cost optical tools, specifically digital VC and NIRI-based vein visualization, can be used to quantify the dynamic responses of microvasculature and superficial veins during a standardized PORH protocol, with PPG serving as a reference technique. We hypothesized that limb occlusion would produce detectable, quantifiable changes in OxyHb-related image features in VC recordings and that superficial venous dilation patterns would be associated with microvascular perfusion dynamics, potentially reflecting venous compliance and contributing to the broader physiological understanding of PORH.

## Materials and methods

### Participants

Fourteen healthy volunteers (21.5 ± 4.2 years old) participated in this study after providing informed written consent. All participants met the predefined inclusion and exclusion criteria. The inclusion criteria were male or female (non-pregnant), between 18 and 35 years old, non-obese (body mass index < 30 kg/m^2^), and non-hypertensive (blood pressure < 130/90 mmHg). The defined exclusion criteria were the current or history of cardiovascular, metabolic, neurologic, or psychiatric diseases, taking vasoactive medications (except contraceptives) or dietary supplements, and tobacco smoking. Participants were instructed to avoid caffeinated beverages and physical exercise for 12 h prior to the procedures. The characteristics of the assessed subjects are presented in Table 1.

**Table 1:** Characteristics of the sample data subjects, expressed in mean ± standard deviation

	Total	Females	Males
N	14	8	6
Age	21.5 ± 4.2	21.5 ± 1.2	21.0 ± 6.4
Height (m)	1.73 ± 0.1	1.69 ± 0.1	1.75 ± 0.1
Body mass (kg)	63.0 ± 8.5	61.0 ± 6.0	65.0 ± 10.5
Body mass index (kg/m^2^)	23.1 ± 2.3	23.2 ± 2.1	21.0 ± 3.0
Systolic blood pressure (mmHg)	111.0 ± 8.5	107.5 ± 8.7	114.0 ± 6.8
Diastolic blood pressure (mmHg)	74.0 ± 6.7	76.0 ± 7.4	74.0 ± 5.7
Menstrual cycle duration (days)	-	28 ± 5	-
Menstrual cycle day	-	8 ± 15	-

### Experimental procedure

The study was approved by the Ethics Committee for Human Research (CEISH) of the School of Pharmacy of the University of Lisbon and conducted in accordance with the Declaration of Helsinki and its subsequent amendments for research involving human participants [12]. All measurements were performed in a temperature- and humidity-controlled room (23–25 °C, 40–60%). Participants acclimatized to the environment in a seated position for 20 min prior to the procedure and were asked to empty their bladders beforehand. They remained seated upright, wearing light clothing, with both hands placed prone on a tabletop at heart level. A pressure cuff was applied to a randomly selected arm, approximately 2 cm above the elbow. The protocol comprised three phases: (I) a 5-min resting baseline; (II) a 3-min occlusion phase with the cuff inflated to 200 mmHg; and (III) a 5-min recovery phase following rapid cuff deflation.

### Technologies

Two reflection PPG sensors were fitted into the palmar aspect of the distal phalanx of the index fingers of both hands. The sensors emitted a green light with a 530 nm wavelength and had a 2.3 mm separation between the LED and receptor. Photoplethysmography quantifies light reflected from the microvasculature, offering an indirect estimate of blood volume [13]. The PPG waveform is composed of two components: a direct current (DC) and an alternating current (AC). The DC component corresponds to the amount of signal reflected by the illuminated tissues, which depends on the amount of blood flow contained in blood vessels, as well as on the optical characteristics of the tissue itself. The AC component, however, is related to the changes in blood volume created by arterial pulses that follow each cardiac cycle. Recent studies have proposed that the PPG waveform is also explained by changes in the capillary diameter caused by fluctuations in arterial transmural pressure [14], and by changes in the red blood cell orientation [15] and deformation [16] in these capillaries.

Skin temperature was recorded to determine whether changes in blood flow were associated with thermoregulatory processes, as suggested by guidelines of cutaneous perfusion quantification [17]. Two negative-temperature coefficient NTC thermistors were fixed to the middle phalanx of the index fingers of both hands. The PPG and temperature sensors were connected to a BITalino Plugged Revolution® microprocessor board (Plux Biosignals, Portugal). Signals were acquired at a 100 Hz sampling rate.

Videocapillaroscopy was used to quantify perfusion based on the image analysis of skin capillaries. The digital microscope (Shenzhen Kingmax Technology Co. Ltd, Guangdong, China) was connected to a laptop via a USB cable. The light source consisted of eight light-emitting diodes (LED) lights at approximately 2 cm to the nailfold. The light intensity emitted by the LEDs was kept constant in all subjects. The microscope’s objective housing was fixed to the nailfold region of the fourth digit on the test hand. Microscopy images were obtained at approximately 120 × magnification and were continuously recorded throughout the entire procedure, generating a video file for each subject.

A NIRI device equipped with near-infrared LEDs was used to visualize superficial veins of the hand dorsum. Hemoglobin from the circulating red blood cells absorbs considerably higher levels of the emitted near-infrared light in comparison with the surrounding skin. The connected computer processes differential absorption and reflection of light, forming an image of the venous courses. A (ZY500 Vein Display, Shenzhen ZY Technology Co., Ltd., Guangdong, China) was used to visualize the superficial veins of the hand dorsum. The device emits low-energy near-infrared radiation toward the skin, which is preferentially absorbed by deoxyhemoglobin in the superficial veins, whereas the surrounding skin absorbs significantly less light [18]. The device detects the differential reflection of light by these structures and generates a contrast-enhanced image, which is then projected on a computer. The LEDs were placed approximately 30 cm above the subjects’ hand, and images were acquired continuously throughout the entire procedure, again generating a video file for each subject.

### Analysis

Three time intervals were defined for statistical analysis: 2–5 min (baseline), 5–8 min (occlusion), and 8–11 min (hyperemia). Skin blood flow was estimated as the amplitude of the PPG waveform (i.e., value at the systolic peak of a given pulse wave minus the value at the onset point of the following pulse wave) and was expressed in arbitrary units (AU). Pulse (min^−1^) was calculated as the number of PPG pulse waves per minute from the contralateral hand PPG signal. Skin temperature values were initially acquired by analog-to-digital converters ADC and converted to their respective degrees Celsius according to the manufacturer’s specifications. Videocapillaroscopy recordings were processed in MATLAB R2015a (MathWorks, USA) and segmented into individual frames. The size of each frame was reduced to 75% of its original dimensions, and the number of frames was downsampled by a factor of 400 to ensure efficient processing time. Each frame was separated into its red (R), green (G), and blue (B) color channels. Changes in intensity in the green and blue planes are largely caused by changes in the concentration of hemoglobin and melanin, while changes in the red plane mainly depend on changes in the concentration of melanin. From these channels, the hemoglobin index (C_Hb_), a parameter related to oxyhemoglobin saturation, was calculated throughout the procedure for each pixel with the following equation, as provided by Tesselar and Farnebo (2021), and expressed in AU [19]:

$${C}_{Hb}=0.483.\text{log}\left(R\right)-0.574.\text{log}\left(G\right)+0.136.\text{log}(B)$$

The percent change between the occlusion and baseline (ΔII-I) and between recovery and baseline (ΔIII-I) was calculated for all variables. Additionally, for the PPG and C_Hb_ signals, several quantitative parameters were calculated, adapted from the classic PORH parameters assessed for the LDF signals (Table 2) [2].

**Table 2:** List of the quantitative parameters obtained from the PPG and C_Hb_ signals

Parameter	Description
*Min*	Minimum PPG amplitude or C_Hb_ value recorded during the occlusion phase (AU)
*t*_*min*_	Time to min—time from the beginning of the occlusion to the minimum skin blood flow during the occlusion phase (s)
*Peak*	Maximum PPG amplitude or C_Hb_ value recorded in the first seconds of the hyperemic phase (AU)
*t*_*peak*_	Time to peak—time from the end of the occlusion phase to the maximum PPG amplitude or C_Hb_ value recorded in the first seconds of the hyperemic phase (s)
*Max*	Maximum PPG amplitude or C_Hb_ value recorded during the entire hyperemic phase (AU)
t_max_	Time-to-max—time from the end of the occlusion phase to the absolute maximum value (s)
*dV*	Decrement velocity— average rate of decrease of the PPG or C_Hb_ value (between 5 and 8 min) (AU/min)
*iV*	Increment velocity— average rate of increase of the PPG or C_Hb_ value (between 8 min and time at peak value) (AU/min)

The video recordings obtained from the vein finder device were uploaded to MATLAB and decomposed into their respective frames. After downsampling the number of frames by a factor of 400, three veins were selected for further analysis. For each vein, the width of three segments (one proximal, one median, one distal) was measured on minutes 1, 3, 5, 8, 11, and 13 with ImageJ v1.54g (National Institutes of Health, Bethesda, MD, USA). The width was calculated for each phase (baseline, minutes 1 and 3; occlusion, minutes 5 and 8; hyperemia, minutes 11 and 13).

The Shapiro–Wilk test revealed that most variables were normally distributed. However, given the relatively small sample size, nonparametric tests were used to ensure analysis robustness. For each phase, all variables were defined as the medians and the corresponding limits of the 95% confidence intervals. Phase comparisons and limb comparisons were carried out using the Wilcoxon signed-rank test for related samples. We hypothesized that the magnitude of venous congestion was related to the magnitude of microvascular perfusion decrease. Therefore, linear correlations between the venous and microvascular perfusion parameters were tested with the Spearman’s Rho coefficient. For all statistical analyses, a 95% confidence level was used. Statistical analyses were performed with SPSS (version 21.0, IBM Corp, Chicago, IL, USA).

## Results

**Fig. 1:**
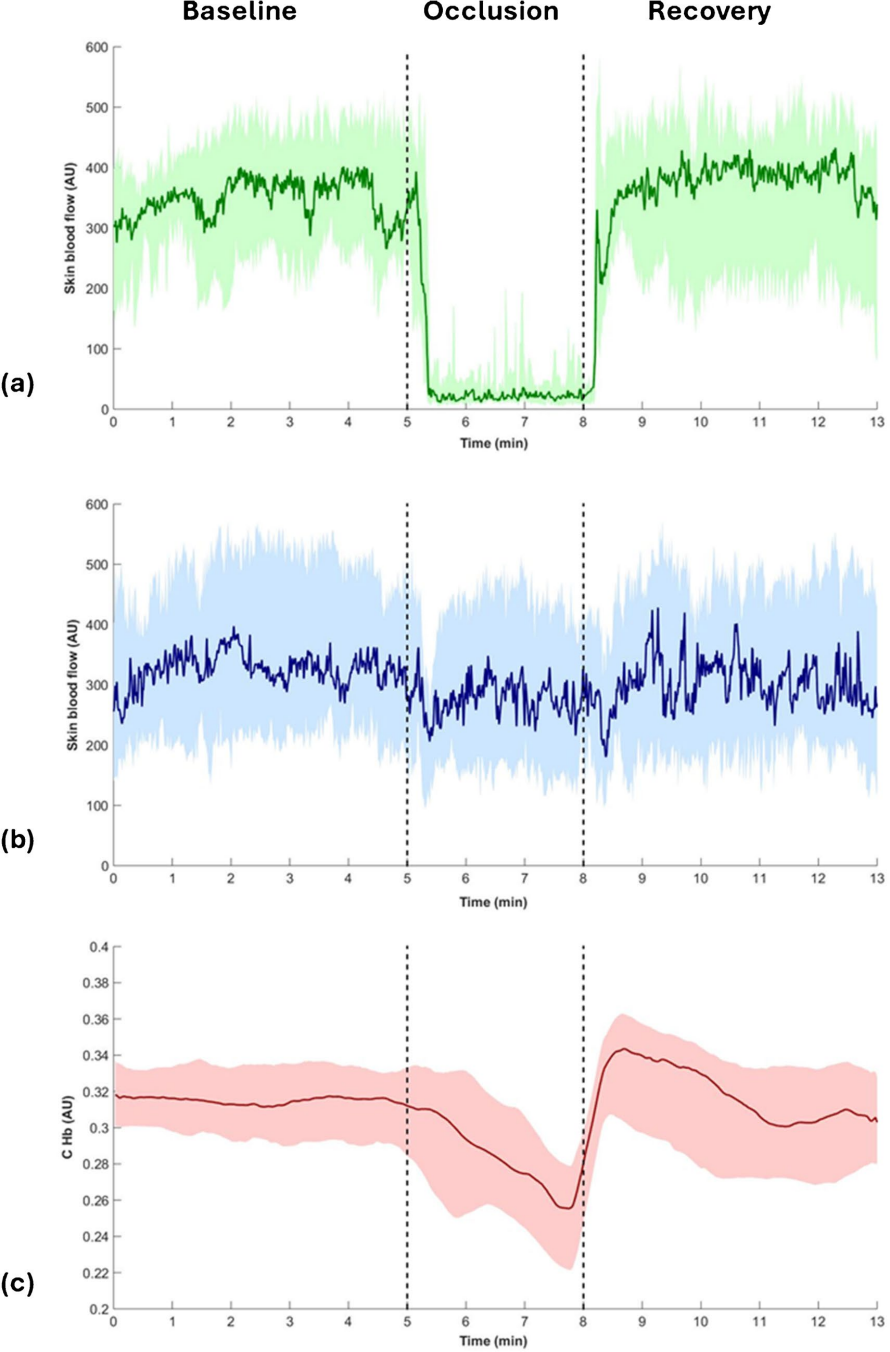
Median (*N* = 14) profile and interquartile range (Q1–Q3) of the perfusion variables obtained throughout the procedure: skin blood flow obtained by PPG in the test hand (**a**) and contralateral hand (**b**) and C_Hb_ obtained by VC (**c**). The shaded areas represent the interquartile range, while solid lines indicate the group median. Vertical dashed lines represent the beginning and end of the occlusion phase (5–8 min)

The graphical representations of the evolution of median and interquartile range of skin blood flow and C_Hb_ are presented in Fig. 1. The medians and 95% confidence intervals of the main variables in each phase of the procedure are presented in Table 3. During the baseline phase, no significant differences in skin blood flow or temperature were observed between limbs, indicating a uniform perfusion distribution. As expected, occlusion caused a significant decrease in skin blood flow (− 95.3%, *p* = 0.001) and in hemoglobin index (− 8.3%; *p* = 0.007) in the test hand. Interestingly, in the contralateral hand skin blood flow also decreased significantly (− 15.0%, *p* = 0.009). Skin blood flow remained significantly lower in the test hand (*p* = 0.001) and reached a significantly lower minimum value (*p* = 0.001) than the contralateral hand, although at a statistically similar median time. Skin temperature decreased in both hands, with significantly higher magnitude in the test hand (*p* = 0.002), although reached values were not significantly different than baseline. Despite this, temperature remained significantly lower in the test hand (*p* = 0.004) during this phase. Vein width increased significantly during occlusion (10.2%; *p* = 0.003). Cuff release elicited a hyperemic response characterized by an initial low-amplitude peak, followed by a nadir and a gradual increase to a maximum over the subsequent 90 s. The median peak amplitude was not significantly different from the median amplitude during the resting phase, although the median value of the maximum was significantly higher. The maximum skin blood flow was reached at 77.3 s before stabilizing. During the recovery phase, no significant perfusion differences were observed when compared to the resting phase in either hand. Temperature decreased further, being more pronounced in the test hand (*p* = 0.001) and was significantly lower than baseline (*p* = 0.004). In contrast, the contralateral temperature was not different than baseline. Vein width returned to its baseline value during recovery. Pulse did not change significantly throughout the protocol.

**Table 3:** Median and 95% confidence intervals for the main quantitative parameters used in the suprasystolic occlusion protocol (*p < 0.05)

Parameter	Test hand	Contralateral hand	*p*-value (test vs. contralateral)
***Skin blood flow***			
Baseline (AU)	327.3 (217.5; 437.1)	355.8 (237.8; 473.8)	0.397
Occlusion (AU)	18.0 (5.2; 30.8)	303.5 (194.3; 412.7)	0.001*
Recovery (AU)	326.8 (219.5; 434.0)	344.8 (233.2; 456.4)	0.594
Δ II-I (%)	− 95.3 (− 98.1; − 92.5)	− 15.0 (− 29.6; − 0.4)	0.001*
Δ III-I (%)	− 13.0 (− 27.5; 1.5)	− 6.6 (− 21.5; 8.3)	0.925
*p*-value (occlusion vs. baseline)	0.001*	0.009*	-
*p*-value (recovery vs. baseline)	0.158	0.084	-
Min (AU)	3.0 (0.8; 5.2)	113.8 (63.6; 164.0)	0.001*
t-min (s)	126.0 (85.7; 166.3)	50.3 (6.8; 93.8)	0.331
peak (AU)	328.0 (201.6; 454.4)	-	-
t-peak (s)	18.0 (13.3; 22.7)	-	-
Max (AU)	467.0 (316.0; 618.0)	-	-
t-max (s)	77.3 (44.8; 109.7)	-	-
dV (AU/min)	− 175.5 (− 260.0; − 91.2)	-	-
iV (AU/min)	1390.8 (720.1; 2061.4)	-	-
***Hemoglobin index***			
Baseline (AU)	0.31 (0.29; 0.34)	-	-
Occlusion (AU)	0.29 (0.26; 0.31)	-	-
Recovery (AU)	0.33 (0.30; 0.36)	-	-
Δ II-I (%)	− 8.3 (− 15.1; − 1.5)	-	-
Δ III-I (%)	1.7 (-4.1; 7.4)	-	-
*p*-value (occlusion vs. baseline)	0.007*		-
*p*-value (recovery vs. baseline)	0.177		-
Min (AU)	0.24 (0.21; 0.27)	-	-
t-min (s)	174.3 (149.0; 199.5)	-	-
Max (AU)	0.35 (0.32; 0.39)	-	-
t-max (s)	26.4 (15.7; 37.0)	-	-
dV (AU/min)	− 0.03 (− 0.04; − 0.02)	-	-
iV (AU/min)	0.22 (0.11; 0.32)	-	-
***Skin temperature***			
Baseline (°C)	32.8 (31.7; 33.7)	32.9 (32.0; 33.6)	0.197
Occlusion (°C)	32.4 (31.6; 33.1)	32.6 32.1; 33.2)	0.004*
Recovery (°C)	32.1 (31.2; 33.0)	32.6 (32.0; 33.3)	0.002*
Δ II-I (%)	− 1.3 (− 2.5; − 0.1)	− 1.3 (− 2.5; − 0.1)	0.002*
Δ III-I (%)	− 2.1 (− 3.1; − 1.1)	− 0.4 (− 1.5; 0.7)	0.001*
*p*-value (occlusion vs. baseline)	0.139	0.878	-
*p*-value (recovery vs. baseline)	0.004*	0.284	-
**Pulse**			
Baseline (min^−1^)	-	75.1 (68; 83)	-
Occlusion (min^−1^)	-	73.3 (64; 82)	-
Recovery (min^−1^)	-	76.2 (68; 84)	-
Δ II-I (%)	-	− 0.9 (− 4.0; 2.0)	-
Δ III-I (%)	-	0.5 (− 2.0; 3.0)	-
*p*-value (occlusion vs. baseline)	-	0.233	-
*p*-value (recovery vs. baseline)	-	0.451	-
**Vein width**			
Baseline (mm)	3.7 (3.4; 4.0)	-	-
Occlusion (mm)	4.0 (3.6; 4.4)	-	-
Recovery (mm)	3.9 (3.5; 4.3)	-	-
Δ II-I (%)	10.2 (5.1; 15.3)	-	-
Δ III-I (%)	3.5 (0.4; 6.5)	-	-
*p*-value (occlusion vs. baseline)	0.003*	-	-
*p*-value (recovery vs. baseline)	0.746	-	-

A statistical comparison of the key quantitative parameters derived from VC and PPG is presented in Table 4. The occlusion produced a significantly higher perfusion reduction in PPG (*p* < 0.001), as well as a higher decrement velocity (*p* < 0.001), whereas the time to reach the minimum perfusion was significantly higher in VC (*p* = 0.001). During recovery, however, the rate of perfusion increase showed no differences between techniques. The time to reach the maximum perfusion value (*p* = 0.002) and the increment velocity (*p* < 0.001) were significantly higher in PPG.

**Table 4:** Statistical comparison between the most relevant PPG and VC parameters (*p < 0.05)

Parameter	PPG	VC	*p*-value
Δ II-I (%)	− 95.3 (− 98.1; − 92.5)	− 8.3 (− 15.1; − 1.5)	< 0.001*
Δ III-I (%)	− 13.0 (− 27.5; 1.5)	1.7 (− 4.1; 7.4)	0.077
t-min (s)	126.0 (85.7; 166.3)	174.3 (149.0; 199.5)	0.001*
t-max (s)	77.3 (44.8; 109.7)	26.4 (15.7; 37.0)	0.002*
dV (AU/min)	− 175.5 (− 260.0; − 91.2)	− 0.03 (− 0.04; − 0.02)	< 0.001*
iV (AU/min)	1390.8 (720.1; 2061.4)	0.22 (0.11; 0.32)	< 0.001*

The Spearman’s statistical correlation between the variation of vein width and other perfusion parameters is presented in Table 5. The percent change in vein width was not significantly correlated with any perfusion parameter derived from VC. However, the percent change between recovery and baseline showed a significant positive correlation with the decrement velocity calculated from PPG.

**Table 5:** Spearman’s linear correlation between relevant perfusion parameters (*p < 0.05)

	Parameters		VC				PPG			
			Δ II-I (%)	Δ III-I (%)	dV (AU/min)	iV (AU/min)	Δ II-I (%)	Δ III-I (%)	dV (AU/min)	iV (AU/min)
NIRI	Δ II-I (%)	Rho	0.136	− 0.418	− 0.178	0.314	0.064	− 0.536	− 0.064	− 0.145
		*p*-value	0.689	0.201	0.601	0.346	0.853	0.089	0.853	0.670
	Δ III-I (%)	Rho	0.191	0.409	0.103	0.264	-0.236	0.255	0.664	− 0.536
		*p*-value	0.574	0.212	0.763	0.432	0.484	0.450	0.026*	0.089

## Discussion

### PORH mechanisms

The mechanisms underlying reactive hyperemia are complex and not yet fully understood. The large increase in perfusion upon cuff release is generally explained by the flow of blood accumulated proximally to the occlusion and by the dilation of vessels distally to the occlusion. The vasodilation itself is complex and explained by several mechanisms. Ischemia enhances nitric oxide (NO) production through increased endothelial nitric oxide synthase (eNOS) activity and reduced NO degradation in hypoxic conditions [20]. Also, the switch to anaerobic metabolism increases the release of carbon dioxide, lactate, and hydrogen ions, which also induce vasodilation [21]. Reduced blood flow during ischemia leads to decreased distension of arterial and arteriolar walls, triggering vascular smooth muscle relaxation, a mechanism known as myogenic dilation [22]. Finally, sensitive nerves also release vasodilator mediators [23, 24], although their contribution to reactive hyperemia is still far from completely understood.

### PPG signal

During occlusion, the PPG amplitude did not reach absolute zero but remained slightly above it, as observed with other techniques such as LDF [25]. This residual signal, termed the “biological zero” is attributed to the movement of trapped red blood cells under illumination [26]. The reactive hyperemia profile observed in PPG resembles that of pulse-wave tonometry [1], with an initial low-amplitude peak occurring in the first seconds, after which blood flow continues to increase until a maximum value is reached several seconds later. This profile is in sharp contrast to the one described for other techniques, such as LDF, where a maximum value is immediately obtained upon cuff release.

### VC signal

Nailfold capillaroscopy is largely used in the clinical setting to assess the morphology of capillaries as an adjunct to the clinical diagnosis of autoimmune diseases such as systemic sclerosis and Raynaud phenomenon [27]. In contrast, NFC is not routinely used to describe the microvascular response to occlusion and PORH. One study described the changes in functional capillary density in patients with pulmonary hypertension after PORH [28]. A few studies have used VC (i.e., as continuous recordings) to investigate perfusion in reactive hyperemia protocols [29–33]. These authors used VC to assess red blood cell velocity in a single capillary when occlusion was applied to a finger [30]. However, assessing perfusion with hemoglobin index, similar to polarized light spectroscopy [34], has not been attempted before with VC. Also, the effects of the whole limb occlusion have not been recorded with VC in the finger.

**Fig. 2:**
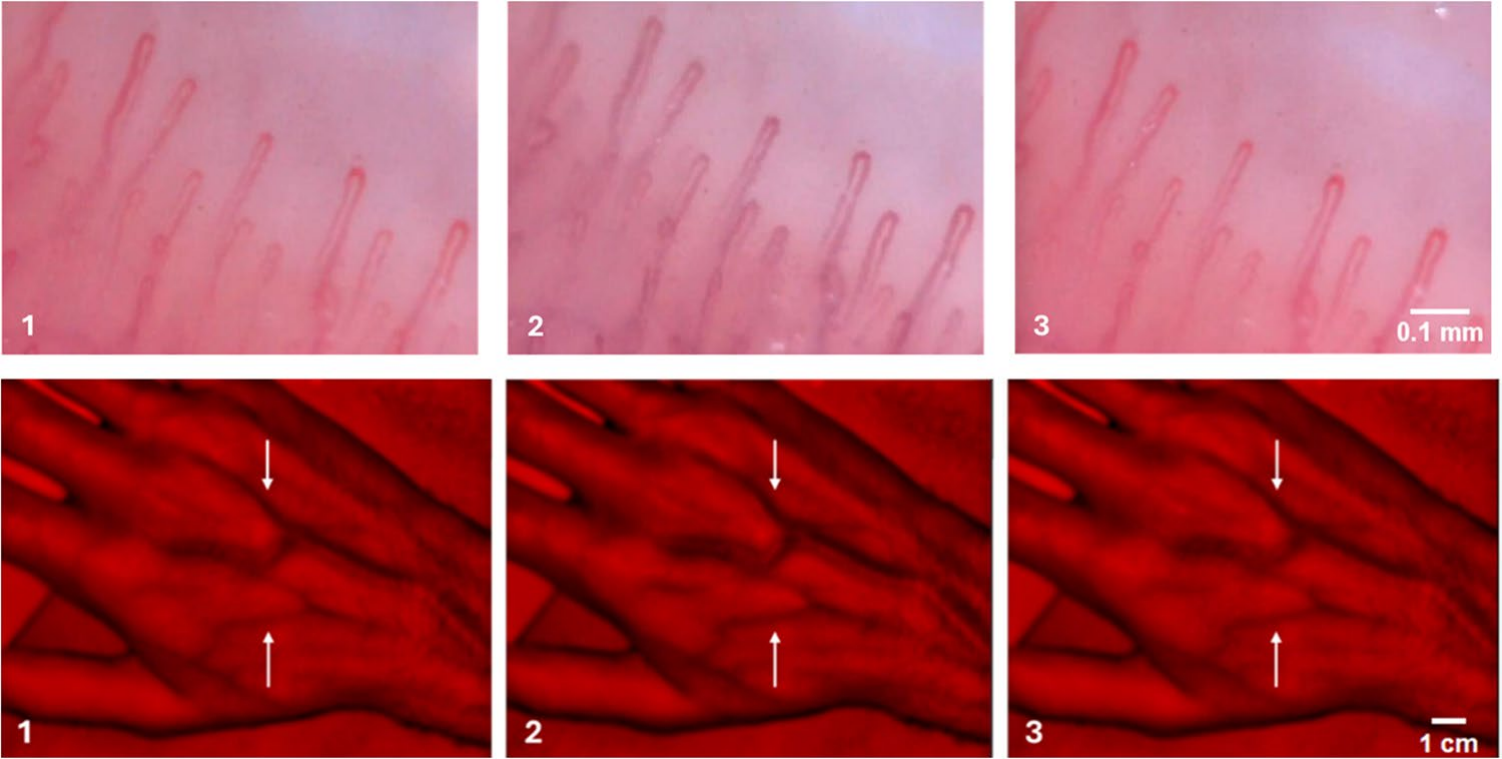
Videocapillaroscopy photos (~ 120 × magnification) and NIRI vein images from a representative subject (**1**, baseline; **2**, occlusion; **3**, recovery). The arrows point to subtle changes in venous width.

The VC frames acquired with the low-cost digital microscope showed good quality overall (Fig. 2). At a magnification capacity of ~ 120 × , an upper row of capillary hairpin loops was clearly visible, running parallel to the length axis of the finger. The time course of the C_Hb_ throughout the procedure is similar to the PPG profile. Upon occlusion, a marked decrease in C_Hb_ was also observed, and, upon cuff release, a sharp increase was also observed. The changes in the C_Hb_ are explained by the changes in the content of OxyHb, resembling the NIRS signal. During occlusion, oxygen continuously diffuses down its partial pressure gradient from capillaries to the surrounding tissues. Since occlusion prevents oxygen renewal, there is a gradual decrease in the OxyHb content in the red blood cells trapped in the capillaries. Conversely, carbon dioxide diffuses from the tissues to the capillaries also down its partial pressure gradient. As such, the levels of both deoxyHb and carbaminohemoglobin (CarbHb) increase, with the latter imparting a bluish discoloration, creating cyanosis. Upon cuff release, the capillary loops are abruptly perfused with blood containing a considerably higher level of OxyHb, which leads to the restoration of the original pinkish coloration.

Our findings reveal a marked difference between VC and PPG in their responsiveness to hemodynamic changes. The percent reduction in C_Hb_ was considerably smaller than the percent reduction in skin blood flow estimated with PPG. Several factors could explain this discrepancy. Firstly, VC and PPG operate under distinct biophysical principles. PPG assesses both arterioles, capillaries, and venules of the cutaneous superficial plexus, whereas VC only assesses the upper row of functional (i.e., perfused) capillaries of the superficial plexus. Furthermore, there is a considerably larger density of arteriovenous anastomoses in the finger pulp, which carry a higher sympathetic innervation [35], than in the finger dorsum. As such, under the present conditions, VC and PPG capture different aspects of perfusion, and VC likely underestimates overall cutaneous blood flow. Secondly, the fact that VC only assesses functional capillaries may explain the lower dV and iV as well as the higher *t*_min_ values following occlusion, as more time is needed to recruit previously closed capillary loops and vice versa. Thirdly, these discrepancies may reflect some degree of regional heterogeneity in the development of reactive hyperemia, which may not be detected by VC. Finally, since red blood cells remain oxygenated at the onset of occlusion, the slower decline in C_Hb_ may reflect residual tissue oxygen reserves. Even though our data suggests that VC is considerably less sensitive than PPG for assessing perfusion, it seems to complement that technique.

### Contralateral perfusion

Our results show that suprasystolic limb occlusion creates a significant contralateral perfusion decrease, which mirrors previously published studies. In fact, these studies have consistently demonstrated that provocations applied to one limb significantly impact the contralateral limb, either the upper or the lower [36–38]. In the specific case of suprasystolic upper limb occlusion, a significant decrease in contralateral perfusion has been observed, as assessed with LDF and PPG [36]. This contralateral perfusion reduction is most likely mediated by sympathetic vasoconstriction, potentially triggered by multiple mechanisms. The perception of discomfort stemming from the limb occlusion may trigger a centrally-mediated increase in sympathetic vascular tone [39]. It is also possible that the venous congestion distal to the occlusion site might evoke this contralateral response, as it was also observed to accompany the venoarteriolar reflex [37]. Future studies should assess perfusion in distal regions to assess the impact of sympathetic activation in the perfusion of other organs.

### Vein finders

In recent years, there has been a surge in interest in “vein finders”, compact devices that highlight the course of superficial veins in the body and which are mainly used as teaching tools to assist venipuncture [9–11]. Some devices project the course of the veins back onto the skin, therefore directly guiding the procedure. Other devices, such as the one used in this study, project the vein course image onto a computer screen. Preliminary experiments showed a ~ 2 s lag in the display of the dorsal hand veins. This latency suggests a slight processing delay, dependent on data transmission speed. Nevertheless, this time offset is very small and, therefore, does not affect the detection of important time points regarding hemodynamic changes in this procedure.

It is worth noting that in a previous study using the same experimental protocol, the authors also reported the superficial vein width value expressed in millimeters [40]. However, those values were obtained from magnified images without spatial calibration and should be interpreted as image-derived representations of venous width, rather than anatomical diameters. In NIRI, the visualized vein contour is influenced by optical scattering, absorption gradients, and subcutaneous tissue properties, generating a peripheral blur or optical penumbra. As such, the measured “width” in that study reflected both the vessel and its surrounding diffused signal, potentially overestimating true lumen dimensions. In contrast, both the present study and a recent publication using the same imaging modality and protocol [41] applied a calibrated pixel-to-millimeter conversion to derive anatomically meaningful vein diameters. Although some degree of optical spread remains inherent to the technique, spatial calibration enables more accurate and physiologically interpretable measurements.

Three segments from three distinct veins were assessed for width at discrete time points throughout the procedure. Occlusion decreased venous return from the hand and forearm regions of the test limb, forcing the blood to accumulate in the venous system distal to the pneumatic cuff. Blood congestion caused an enlargement in the veins of the hand dorsum, resulting in a significant increase in venous width. Upon release of the cuff, venous return increased rapidly, leading to a resumption of the initial venous caliber. The positive correlation between venous diameter increase and microvascular perfusion decrement velocity suggests a shared hemodynamic response between arterial and venous compartments during PORH. Arterial occlusion reduces perfusion pressure while concurrently causing venous congestion distal to the cuff, both of which contribute to a reduced capillary-to-venous pressure gradient. In this context, a greater reduction in microvascular flow velocity appears to indirectly reflect higher venous compliance, as increased venous dilation may facilitate passive blood accumulation, altering capillary drainage dynamics upon cuff release.

### Comparison of perfusion assessment techniques

Several optical and ultrasound-based techniques have been developed to assess peripheral perfusion and vascular reactivity. Each method has specific advantages and limitations that should be considered when selecting a tool for clinical or research purposes.

Doppler-based optical techniques allow the quantification of blood flow. Laser Doppler flowmetry provides continuous and highly sensitive measurements of skin blood flow with excellent temporal resolution, although it is limited to small skin areas, which may reduce its feasibility for routine or point-of-care applications [25]. Laser Doppler imaging (LDI), in contrast, offers transversal perfusion measurements across larger skin surfaces [42]. Laser speckle contrast imaging (LSCI) enables full-field perfusion imaging and has gained popularity due to its capacity to capture spatial heterogeneity in skin microcirculation [43]. However, LSCI is affected by surface geometry and has relatively lower sensitivity to perfusion changes in deeper tissue layers. All laser Doppler techniques tend to be expensive and are particularly susceptible to motion artifacts. Near-infrared spectroscopy estimates tissue oxygenation and hemodynamics in deeper vascular compartments, providing valuable insights into both arterial and venous physiology [44]. Nevertheless, NIRS signals can be influenced by subcutaneous fat thickness and require cautious interpretation due to the composite nature of the recorded signal. Tissue viability imaging is another non-invasive optical technique based on polarized light spectroscopy, which quantifies changes in red blood cell concentration in the skin [45]. It allows wide-field, map-like visualization of perfusion and has been validated in several vascular reactivity protocols. While highly effective, TiVi systems remain relatively costly and are not yet widely adopted outside research settings. Ultrasound Doppler techniques, including color and spectral Doppler, are widely available in clinical settings and allow the assessment of flow velocity and vessel diameter in both superficial and deep vessels [46]. Despite their utility, these techniques are highly operator-dependent, require specific training, and are less suited for continuous monitoring in dynamic protocols such as PORH.

In contrast, the techniques employed in the present study, PPG, VC, and NIRI, offer distinct advantages in terms of portability, cost-effectiveness, and ease of implementation. Photoplethysmography enables continuous monitoring of pulsatile blood flow with minimal equipment and is highly sensitive to changes in perfusion. Videocapillaroscopy provides direct visualization of capillary morphology and oxygenation-related color changes, while NIRI allows real-time assessment of superficial venous dynamics. Although these methods may offer lower spatial or temporal resolution compared to more advanced systems, their combined use provides a practical and accessible approach to assess both microvascular and venous responses during reactive hyperemia, especially in resource-limited or ambulatory settings.

## Study perspectives and limitations

This study offers promising perspectives for the advancement of non-invasive vascular assessment methods. The quantification of perfusion using low-magnification VC and NIRI highlights the potential of portable, low-cost technologies for application in both research and clinical settings. These techniques may be particularly valuable for the early detection of microvascular dysfunction in conditions such as hypertension, diabetes mellitus, peripheral vascular disease, and systemic sclerosis. Future studies could extend the use of VC beyond morphological analysis, establishing it as a functional biomarker of capillary reactivity. The integration of VC and NIRI with automated image processing and machine learning could enhance diagnostic accuracy and reproducibility. The observed correlation between venous dilation and perfusion dynamics suggests that composite indices of vascular compliance may be developed. Additionally, the image datasets obtained may support further research using artificial intelligence for automated analysis and pattern recognition. Validation against gold-standard techniques and longitudinal studies assessing reproducibility and sensitivity to interventions are also warranted.

Nonetheless, several limitations must be acknowledged. First, the relatively small sample size and unbalanced sex distribution limit the generalizability of the findings. Second, the absence of continuous blood pressure monitoring precluded a more comprehensive interpretation of perfusion dynamics. Third, the fasting state of participants was not standardized, which may have influenced vascular reactivity due to variable insulin levels.

## Conclusion

This study provides a methodological contribution to the assessment of PORH. Our results suggest that low-magnification VC can reliably quantify perfusion in vivo. We also demonstrate that superficial vein dimensions can be assessed using NIRI and that venous dilation correlates with the decrement velocity of microvascular perfusion, hinting at the potential to assess venous compliance during PORH. Our findings indicate that these techniques, based on distinct biophysical principles, are complementary and together enable a more comprehensive assessment of PORH.

## Data Availability

The datasets generated and analyzed during the current study are available from the corresponding author upon reasonable request.
